# Geriatric Breast Cancer: Staging, Molecular Surrogates, and Treatment. A Review & Meta-analysis

**DOI:** 10.14336/AD.2023.1002

**Published:** 2024-08-01

**Authors:** Vasco C Fonseca, Zacharoula Sidiropoulou

**Affiliations:** ^1^Department of Oncology, Hospital Centre of West Lisbon, Portugal.; ^2^General Surgery Department, Breast Unit, Hospital Centre of West Lisbon, Portugal

**Keywords:** Breast Cancer, elderly, molecular subtypes, Cancer staging

## Abstract

Breast cancer (BC) is one of the most frequent cancers in females across the globe. Treatment recommendations for BC patients are primarily driven by patient age, staging and tumor molecular subtype. Thus, we updated the general overview of BC staging, molecular surrogates, and treatment choices for women >70 years based on a systematic study encompassing the years 2013-2023. A PRISMA guidelines and PICO framework were followed, and relevant research articles were searched using different data bases (Web of Sciences, PubMed, MEDLINE, and Scopus). Mixed Methods Appraisal Tool was used for studies quality assessment. The research articles that made it into the systematic review were compiled using qualitative criteria. In the meanwhile, heterogeneity was determined using meta-analysis with RevMan 5.4. We applied a random effects model with a 0.05 significance level. Overall, there were 4151 research articles, after screening only 17 articles with 39,906 patients were included. Conclusion: Elderly patients with breast cancer should be treated differently in an adapted way. The treatment should not be the same worldwide due to different health systems. Molecular surrogates are different in geriatric patients. Surgery is the best option for treatment in this subset of patients. We need to have therapeutic decision appointments for elderly patients with breast cancer. The guidelines and medical authority should be used in the best decision.

## Introduction

Within the realm of breast cancer research, a significant gap persists in the comprehensive understanding of disease progression, treatment outcomes, and molecular intricacies in geriatric patients aged 70 and above. While substantial progress has been made in breast cancer diagnosis and treatment, a notable disparity exists in the representation of older women within clinical trials and research endeavors. This study seeks to bridge this gap by shedding light on the unique characteristics of breast cancer in the geriatric population, particularly focusing on staging, molecular profiles, and treatment responses [1]. The importance of addressing this gap is underscored by the growing aging demographic worldwide, where a considerable proportion of breast cancer cases occur in women above 70. Current treatment strategies often stem from studies involving younger patients, potentially leading to suboptimal therapeutic decisions for older individuals.

By elucidating the molecular underpinnings specific to geriatric patients, this study strives to provide valuable insights that can lead to tailored and more effective treatment approaches for this understudied population. Ultimately, the findings from this systematic review and meta-analysis aim to inform clinical practice, enhance treatment outcomes, and improve the quality of life for older women battling breast cancer."

Molecular surrogates refer to specific molecular markers, indicators, or characteristics that are used to indirectly measure or predict certain biological processes, disease states, or treatment responses. In the realm of breast cancer, molecular surrogates often encompass various genetic, genomic, and protein-level factors that provide insights into the underlying molecular mechanisms of the disease. These surrogates are particularly valuable in informing treatment decisions, as they can offer a more nuanced understanding of tumor behavior and potential treatment outcomes [2].

In the case of geriatric patients (those aged 70 and above) with breast cancer, the concept of molecular surrogates takes on added significance due to the unique physiological and biological changes that accompany aging. As individuals age, their bodies undergo various alterations, including changes in hormone levels, immune function, and cellular processes. These age- related changes can influence the behavior of breast cancer at the molecular level, potentially affecting how tumors develop, progress, and respond to treatments.

Moreover, the concept of molecular surrogates becomes especially pertinent in the context of tailoring treatment approaches for geriatric patients. Conventional treatment strategies often draw from data obtained primarily from younger patients, potentially overlooking the unique characteristics of tumors in older individuals. By identifying and understanding molecular surrogates that are relevant to geriatric breast cancer, clinicians can make more informed decisions regarding treatment selection, dosage, and duration. This personalized approach can help mitigate potential adverse effects and optimize treatment efficacy, ultimately improving the quality of life for elderly patients.

The concept of 'molecular surrogates' within the realm of breast cancer research holds particular significance in tailoring treatment strategies. However, when considering the geriatric population, a distinctive void becomes evident. In contrast to the extensive exploration of molecular surrogates in breast cancer characterization, staging, and treatment, a marked scarcity of evidence prevails in international literature, specifically among women aged 70 years and above. The absence of research dedicated to investigating molecular surrogates in this demographic underscores a critical gap in our understanding. This gap is of paramount importance, as the distinct physiological changes that accompany aging can significantly influence the behavior of breast cancer at the molecular level. Consequently, the lack of attention to this age group potentially leads to suboptimal treatment strategies extrapolated from studies on younger patients [1]. Our systematic review, situated within this uncharted territory, aims to rectify this gap by shedding light on the unique molecular landscape of breast cancer in geriatric patients. By identifying and synthesizing available data, our study not only highlights the lack of focus on this claim in international literature but also paves the way for future research that could revolutionize the approach to breast cancer treatment in this often-overlooked patient population.

Breast cancer (BC) is the most frequent cancer among women, worldwide [2], while occurrence and death rates sharply rise with age [3]. Finding the best way to treat breast cancer in elderly women is still a challenge [4]. The annual number of new cases of BC in women is close to 2.3 million, accounting for over half of all cancer deaths in women in over 95% of countries [5]. The most current data years (2010-2019) show a 0.5% annual increase in the incidence rates of BC, mainly because of the prevalence of early detection of hormone receptor-positive illness [6]. Death rates from BC, in contrast, have been gradually falling since their peak in 1989, however, at a lower rate 1.3 % annually from 2011 to 2020 than in the preceding decade 1.9% annually from 2002 to 2011 [7]. Human epidermal growth factor receptor 2 (HER2)-negative, hormone receptor-positive BC was detected early in most female patients. It is estimated that less than one percent of women in their 70s and 80s died from invasive BC due to this disease [7, 8]. Except in patients with severe comorbidities or low life expectancy, screening mammography significantly reduces BC mortality in older women [9]. Moreover, there are the American Cancer Society's recommendations, where patients with "excellent overall health" and with a 10-year or longer life expectancy are encouraged to continue cancer screenings [10,11]. However, the American Academy of Physicians and the American Academy of Family Physicians suggest stopping screening beyond age 74 [12]. Though, American College of Physicians, U.S. Preventive Services Task Force, and American Academy of Family Physicians guidelines recommend screening mammography in women 50-74 years old [13, 14] associated with earlier disease stages, while increased survival is yet to be demonstrated. Women at average risk of BC, aged 40 and up, are now encouraged to get a mammogram every year by the National Comprehensive Cancer Network (NCCN) [15,16]. Similarly, according to ESMO recommendation, mammography is indicated for women 50-69 [I, A]. Although the evidence is weaker, women aged 40-49 and 70-74 may benefit from regular mammography [II, B] [17]. Care providers should consider bringing up the topic of screening mammography with their elderly patients, especially those who don't have any major co-morbidities [18]. Discussions about advantages and hazards, tailored to each woman's health situation, are essential for optimizing screening procedures [9]. Once cancer is a disease of aging, health systems need to adapt healthcare to such patients [19]. When treating BC in older women, it is vital to strike a balance between the treatment's curative purpose and the patient's comorbidities and quality of life [20] Patients in their later years have a decreased life expectancy and a different kind of longevity advantage. Patients >70 years should be evaluated using the global geriatric assessment scale, and scales can be performed by the nursing team [21].

There are gene-profiling models that can predict outcomes. Besides, molecular categorization is the standard for comprehensively characterizing BC the most used being the Oncotype Dx, and Mamaprint; these tests should be used in elderly patients [22-25]. Despite these advances, most doctors still rely on established clinicopathologic features and widely available tumor markers like estrogen receptor (ER), HER2 and progesterone receptor (PR) [26-28]. Additionally, Androgen receptors and p53 are examples of biomarkers that have recently been proven to further stratify these molecular subtypes [28]. Meanwhile, in Western countries at high risk for BC, ER-positive tumors rise in a bimodal fashion, after age 60, luminal A cancers are more common than luminal B tumors with higher proliferation rates [29]. Cancer Centers should have specialized consultations for elderly patients; the optimal treatment might be different from the adequate treatment for this population [30]. Unless there are significant barriers, therapeutic indifference or undertreatment should be avoided, and adequate therapy should be provided [31]. Proactive discussions about treatment with healthcare providers may benefit older women by addressing their unique needs, personalizing cancer care, and easing the healthcare system process [32]. Older women receive less preventative or adjuvant treatment and due to underrepresentation in clinical trials, older women with BC are poorly evaluated and treated [30].

Treatment schedules for healthy women are established, but patients > 75 years are typically not treated according to recommendations due to comorbidities and poor health conditions. Even when cancer is diagnosed at the local or locoregional advanced stage [33, 34], despite some worries and relative contraindications. While reviewing data from the literature and our own experiences, it is crucial to assess how women of advanced age are cared for [35]. It is often up to individual oncologists to make treatment choices in an area of uncertainty for the management of elderly patients with BC. Consequently, this could upsurge the under-treatment risk or, less frequently, overtreatment, which can negatively affect patient outcomes [36]. Alterations in pharmacokinetics and metabolism because of aging are extremely uncommon and it is very important to have the knowledge of current medications to evaluate polypharmacy [37]. Systemic therapy and Adjuvant trastuzumab are preferred over adjuvant systemic therapy alone for older women in good health with HER2-overexpressing BC who have had surgery [38]. Combining HER2-directed therapy with chemotherapy is linked to a modest but real increase in the risk of cardiac dysfunction, especially when an anthracycline-based regimen is used. Therefore, the prognosis of newly diagnosed BC women must be weighed against the associated risk with treatment (combined) [39]. In addition, CGA or miRNA, even if not used in clinical practice, scoring systems could accurately identify patients who need active or palliative treatment [40]. Adjuvant chemotherapy is given after surgery, often between 4 and 8 weeks after the procedure. When treatment is delayed for more than 12 weeks, it may negatively affect disease-free survival; however, earlier therapy does not necessarily result in a better prognosis [41]. Meanwhile according to ESMO guidelines, neoadjuvant treatment should begin 2-4 weeks following diagnosis and staging, while adjuvant treatment begins 3-6 weeks after surgery [17]. Surgery in elderly patient is often the preferred option because it has the least side effects and complications. The elderly patient should be optimized for surgery in a similar way to that prepared by an athlete [17, 42]. Furthermore, several authors propose the use of neo-adjuvant hormonotherapy to evaluate the response to hormonal therapeutics in the patient, this option should be extended in clinical practice [43, 44]. Moreover, immunotherapy can benefit elderly patients, but they also had higher adverse effects and stopped treatment earlier than younger patients [45]. The toxicity of chemotherapy in addition to complications during treatment and difficulty in completing treatment in full, is associated with loss of years of life due to its negative effects of it [45-47], due to the even if not age-related comorbidities and reduction in organ function, elderly individuals have a lower tolerance for chemotherapy as compared to younger patients [48].

Despite the extensive literature on BC treatment, few studies have examined any potential links to molecular surrogates in older women. We conducted a literature review covering the years 2013-2023, and we offered a revised overview of staging, molecular surrogates, and treatment options for BC in women > 70 years.

## METHODS

The current systematic review and meta-analysis was conducted in the line of Preferred Reporting Items for Systematic Reviews and Meta-Analyses (PRISMA) guidelines [49].

### Literature search

**Figure 1.**
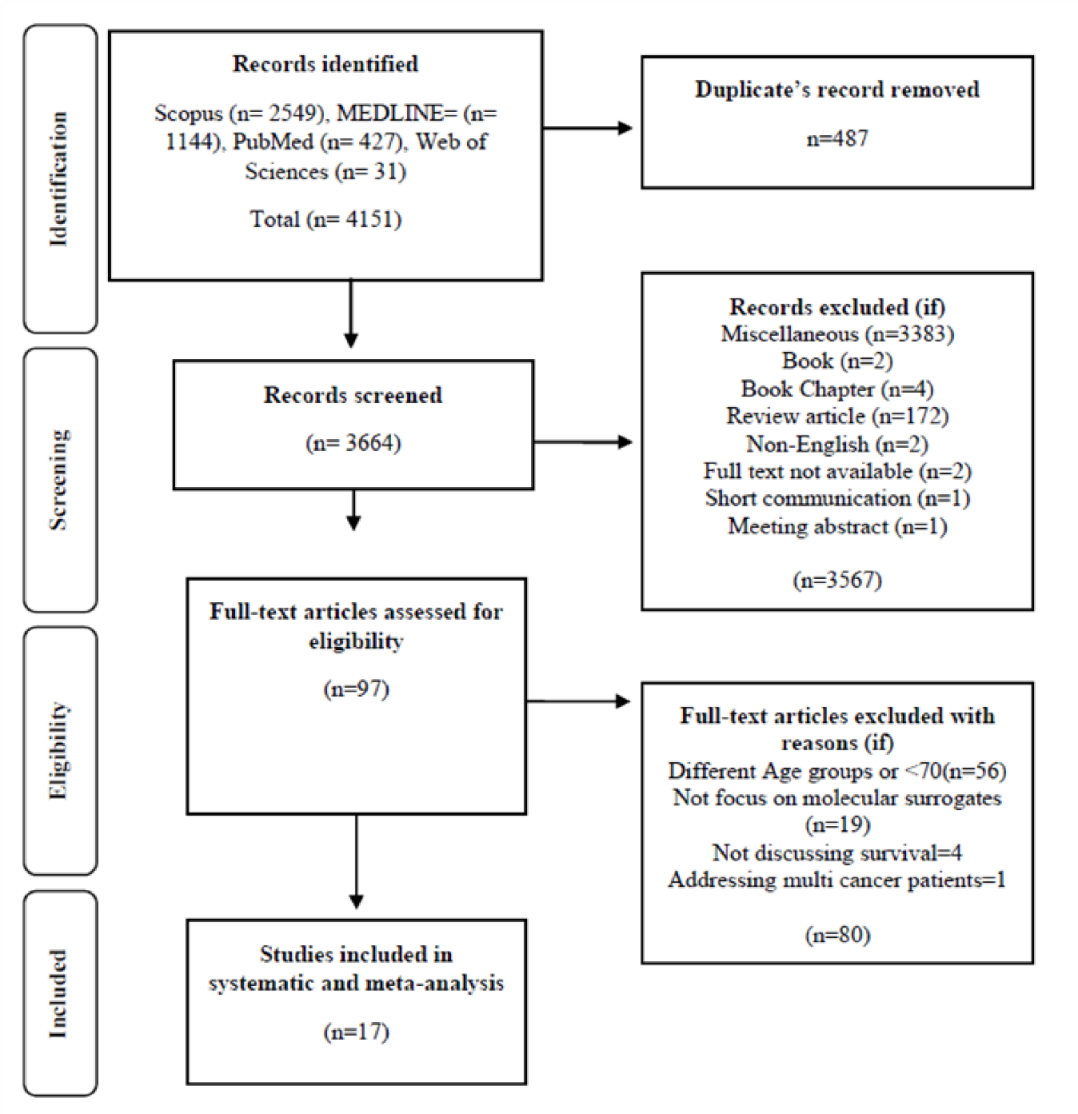
Flowchart for screening of literature.

The search strategy was established according to the participants, intervention, comparators or controls, and outcome (PICO) framework [50]. Population/ Participants: This review included older women (>70 years). Intervention: different treatments were used for BC in elderly women. Comparison or control: Other treatment or control groups used. Outcomes: Updated systematic review and meta-analysis of the literature on the characterization and treatment of BC for elderly women. Different search databases such as Web of Sciences, PubMed, MEDLINE, and Scopus were searched for the relevant research articles using different keywords such as ‘breast cancer’’ and ‘‘older, elder, eldest, elderly’, over 70 years, molecular surrogates, HER2, ER, PR, Ki-67, EGFR, P53, Androgen receptor, hormone receptor-positive and treatment options like chemotherapy, hormonal therapy, radiotherapy, and other medications' MeSH terms were used, Oncotype Dx, Mamaprint , as well as combinations of those terms (Appendix 1) and selected articles were processed according to PRISMA (Fig. 1).

#### Inclusion criteria

The following standards were applied exclusively for selecting studies: 1. Female patients over the age of 70 with a diagnosis of invasive BC (stages I-IV) who were considering or had received different treatments; 2. Studies identifying molecular surrogates; 3. Studies that have been published in peered scientific journals in English; and 4. Studies with patients' perspectives on their experiences with palliative care, alternative medicine, and non-treatment are understudied.

#### Exclusion criteria

The following were not considered for inclusion in the study: 1. Publications that do not undergo the peer review process include editorials, reviews, protocols, guidelines, and journals that are not included in citation databases. 2. Research that doesn't account for patients' ages, treatment histories, or outcomes.

#### Study selection and assessment

Study titles, and abstracts were all assessed separately. Two reviewers independently evaluated the entire texts of all articles that matched the inclusion requirements, whose opinions were then debated to reach a consensus. If there were any discrepancies, they were discussed with the third independent reviewer to resolved.

### Data extraction

Data extraction was done on the shortlisted studies matching the inclusion requirements. A data extraction form was used to record the data that was extracted after screening the paper's title, abstract, and full text. Two reviewers independently record each study's authors, year of publication, mean age, cancer stage, comorbidities, characterization related data, treatment findings, conclusion, and limitations for a systematic review. While for the meta-analysis: total participants, participants in the BC elderly treatment group, and participants in the control group were used to construct forest plots.

#### Quality assessment

Using the Mixed Methods Appraisal Tool (MMAT), quality ratings were determined based on the degree to which each study's methodology met predetermined criteria using the approach described by Charette, McKenna [51]. Studies were classified as either low (scoring ≤3) or high (score >3) depending on the reviewer answered: "yes" (1 point) or "no" (0 points) [52].

#### Data analysis

The included articles in the systematic review were compiled utilizing qualitative analysis. The PRISMA checklist was used to conduct a systematic review of pertinent literature, and a step-by-step method for choosing articles was provided. In the meanwhile, meta-analysis was performed using RevMan 5.4 [53] to calculate the Cochrane Q and *I^2^* values, which quantify trial dispersion. The random effects model was used, with the significance level set at 0.05.

## RESULTS

### Consulted Literature

All research was found in peer-reviewed journals in electronic databases (Scopus, MEDLINE, PubMed, Web of Sciences), which resulted in a total of 4151 relevant publications. There were 487 duplicates that were eliminated. The titles and abstracts of the remaining 3664 papers were then reviewed, and another 3567 were omitted since they were irrelevant to our study. After a thorough evaluation, 80 of the remaining 97 full-text articles were discarded for a variety of reasons (Figure 1). The 17 publications are summarized, and their salient features are listed in tables.

**Table 1:** Qualitative features of included studies.

Ref.	Country	Study design	N	Mean age	Tumor stage (%)					Comorbidities (%)
					0	I	II	III	IV	
[54]	Turkey	Retrospective	83	82	NA	16	41	13	12	Yes
[57]	Singapore	Retrospective	19	84	NA	10.50	36.8 IIa, 26.3 IIb	10.5 IIIa, 15.9 IIIb	0	100
[53]	Turkey	Retrospective	620	75.7	NA	NA	NA	NA	NA	NA
[58]	Chile	Retrospective	366	75.5	NA	32.20	40.10	19.90	7.80	NA
[59]	Canada	Retrospective chart review	762	>70	15.50	50.10	31.90	2.50	NA	NA
[61]	Brazil	Retrospective	70	84	15.40		47.70	33.80	3.10	Yes
[63]	Germany	Retrospective	1292	77.1	NA	NA	NA	NA	NA	29.30
[64]	UK	Retrospective	252	>70	NA	NA	NA	NA	NA	NA
[52]	Turkey	Retrospective	238	75	5	24.80	42.90	27.30	NA	Yes
[62]	Netherland	Retrospective	2390	79.2	NA	NA	NA	NA	NA	Yes
[56]	China	Retrospective	552	>70	NA	NA	NA	NA	NA	Yes
[60]	South Korea	Retrospective	87	74	11.50	29.90	35.60	14.90	8	NA
[55]	China	Retrospective	33,177	77.6	NA	57.70	33.20	9.20	NA	NA

### General characteristics

In the present study, 39.906 patients were reported. Most studies were reported from Turkey [54-56], two studies from China [57, 58] and single studies from Singapore, Chile, Canada, Brazil, Germany, UK, Netherlands, and South Korea. All studies followed a retrospective study design and collect data using national database registries or hospital records. All studies were focused on elderly patients and included patients >70 years’ age. Maximum mean age reported was 84 years [59]. In terms of staging, there were five stages (0-IV) reported by different included studies [54, 56, 57, 59-62] as indicated in Table 1. Meanwhile, seven studies reported comorbidities (Diabetes, Cardiovascular diseases, hypertension) ranging from 29.30% to 100% patients had comorbidities [54, 56, 58, 59, 63-65].

**Table 2:** Molecular surrogates used for the screening of BC in elderly patients.

Ref.	Molecular surrogates (%)								Metastasis status (%)	
	Hormone receptor+	ER Positive	ER Negative	PR Positive	PR Negative	HER2 positive	HER2 Negative	Ki-67	Present	Absent
[54]	NA	73	20	70	20	NA	NA	NA	12.04	NA
[57]	NA	100	0	52.60	NA	10.50	26.30	NA	NA	100
[53]	NA	75	19.30	67.30	26.90	NA	NA	NA	15	NA
[58]	83.30	NA	NA	NA	NA	12.60	NA	NA	42.70	NA
[59]	NA	75.10	24.90	66.90	33.10	18.90	67.20	NA	NA	NA
[61]	NA	76.90	NA	65.20	NA	13.00	NA	NA	15.70	NA
[63]	NA	87.60	12.40	77.40	22.60	16.10	83.90	100	NA	NA
[64]	NA	NA	NA	26.80	73.20	16.90	83.10	70.70	NA	NA
[52]	NA	79	19.30	70.20	28.60	14.30	85.70	NA	NA	NA
[62]	NA	NA	NA	NA	NA	NA	NA	NA	NA	NA
[56]	NA	NA	NA	81.90	NA	NA	81.90	NA	NA	NA
[60]	NA	63.20	24.10	48.30	44.80	21.80	71.30	NA	14.90	75.70
[55]	NA	87.50	12.50	76	23.90	10	90	NA	NA	NA

BC=Breast Cancer; NA=Not Available; ER= Estrogen Receptor; PR=Progesterone Receptor; HER2= Human epidermal growth factor receptor 2; Ki=Kiel

### Molecular surrogates

Among the included studies only one study reported Hormone receptor+ molecular surrogate [60]. Maximum 100% ER positive patients were found by Chan, while 63.20% was the minimum ER positive patients [59]. Maximum ER negative 0% patients were documented and 24.90% was maximum ER negative patients [61]. In terms of PR positive 81.90% was the maximum percentage of patients [58] while minimum PR positive was 26.80% patients [66]. Moreover, 73.20% was the maximum PR negative patients reported by Syed, Morgan [66] and minimum PR negative patients were 20% [56]. Similarly, there were maximum 21.80% HER2 positive patients [62] and 10.50% minimum HER2 positive cases were reported by Chan [59]. Similarly, 90% HER2 negative cases were reported by Wu, Qi [57] while 26.30% was the minimum number of HER2 negative patients [59]. Meanwhile, only two studies reported Ki-67% molecular surrogate [65, 66] (Table 2). Metastasis status of patients is presented in Table 2. Meanwhile, relevant, and advanced surrogates like Oncotype Dx and Mamaprint were not reported in the included studies.

**Table 3:** Subtype reported included studies.

Ref.	Subtype of Breast Cancer			
	Luminal A (%)	Luminal B (%)	Triple negative (%)	HER 2-enriched (%)
[58]	49	33.30	11.70	6
[61]		75.70	20.30	NA
[63]	51	25.80	NA	16.10
[52]	NA	NA	12.10	NA
[60]	21.80	10.30	NA	NA
[55]	81.10	7.10	8.90	2.90

NA=Not Available; HER2= Human epidermal growth factor receptor 2

### Subtype

According to Table 3, there were four different subtypes of BC identified (Luminal A, B, Triple negative, HER2-enriched). Wu, Qi [57] reported maximum 81.10% luminal A subtype and minimum was 21.80% [62]. Similarly, for luminal B 33.30% was the highest percentage of patients [60] while 7.10% was the least number of patients with luminal B [57]. As for Triple negative there was 20.30% highest percentage of patients [63] and 8.90% was the least [57]. HER2-enriched maximum patients (16.10%) were reported by Inwald, Ortmann [65].

### Treatment

All the studies in the present review included patients who were using different therapies such as surgery, chemotherapy, radiotherapy, hormonal/endocrine therapy, adjuvant therapy, neoadjuvant therapy. Surgery was the primary method of treating BC in most of the studies [56, 57, 60, 62, 64-66]. While a single study reported patients using adjuvant and neoadjuvant therapy [54]. The percentage of patients under different treatments with follow-up period are described in Table 4. Meanwhile, in terms of overall survival (OS) 95.2% patients had 7 years of survival [65] and 84.6% patients had Disease Free survival (DFS) (5 years) [60] while 63.2% of patients refused to perform surgery [59]. Furthermore, most of the patients had grade II and III BC while BC was spread to N0 and N1. However, patients with N2 and N3 are also reported (Table 5). Death rate and each study conclusions and limitations are reported in Table 5.

**Table 4:** Different treatments, OS, DFS used by reported patients.

Ref.	Treatment							Follow-up months										
	Total Mastectomy (Surgery) (%)	Axillary Dissection (Surgery) (%)	Radiation (%)	Chemotherapy (%)	Hormonal/Endocrine therapy (%)	Adjuvant therapy (%)	Neoadjuvant therapy (%)		OS (5 years) %	DFS (%)	Sur. Refusal %	Grade (%)			N (%)			
												I	II	III	0	I	II	III
[54]	10	NA	25	16.9	71.08	NA	NA	36	61.9	53.7	NA	16	38	16	30	21	11	5
[57]	5.26	NA	NA	NA	100	NA	NA	28	NA	NA	63.2	NA	NA	NA	NA	NA	NA	NA
[53]	71		Not clear				NA	NA	71-76	NA	NA	NA	NA	NA	NA	NA	NA	NA
[58]	36.6	62.1	79.1	21.1	NA	NA	NA	57.50	76.8	84.6	NA	15.80	39.80	44.40	NA	NA	NA	NA
[59]	NA	Not clear				NA	NA	14	NA	NA	NA	33.4	37.2	29.4	NA	NA	NA	NA
[61]	NA	74.6	3	1.50	20.90	NA	NA	37.1	NA	NA	NA	NA	46.9	36.7	NA	NA	NA	NA
[63]	41.9	NA	26.2	NA	NA	NA	NA	NA	95.2 (7 years)	NA	NA	14.6	63.5	22	NA	NA	NA	NA
[64]	77	NA	5.6%	NA	14.3	NA	NA	37.5	Unclear	NA	NA	4.6	45.4	50	NA	NA	NA	NA
[52]	NA	59.6	53.7	30.6	82.8	87.4	10.5	41.2	NA	NA	NA	16.4	41.6	26.9	47.9	23.9	12.2	7.1
[62]	NA	NA	Unclear	0.16	39.30	NA	NA	57.6	NA	NA	NA	Unclear						
[56]	NA	NA	NA	NA	100	NA	NA	NA	NA	NA	NA	20.83	43.65	17.39	NA	NA	NA	NA
[60]	24.1	2.3	NA	NA	NA	NA	NA	54	NA	NA	NA	Unclear			64.4	17.2	6.9	11.5
[55]	12.3	NA	47.90	15.89	NA	NA	NA	43.7	Unclear	NA	NA	29	46.9	24.1	77	17.1	3.8	2.2

NA=Not Available, OS=Overall Survival, DFS=Disease Free Survival, N=Node

### Sub-group analysis

**Figure 2.**
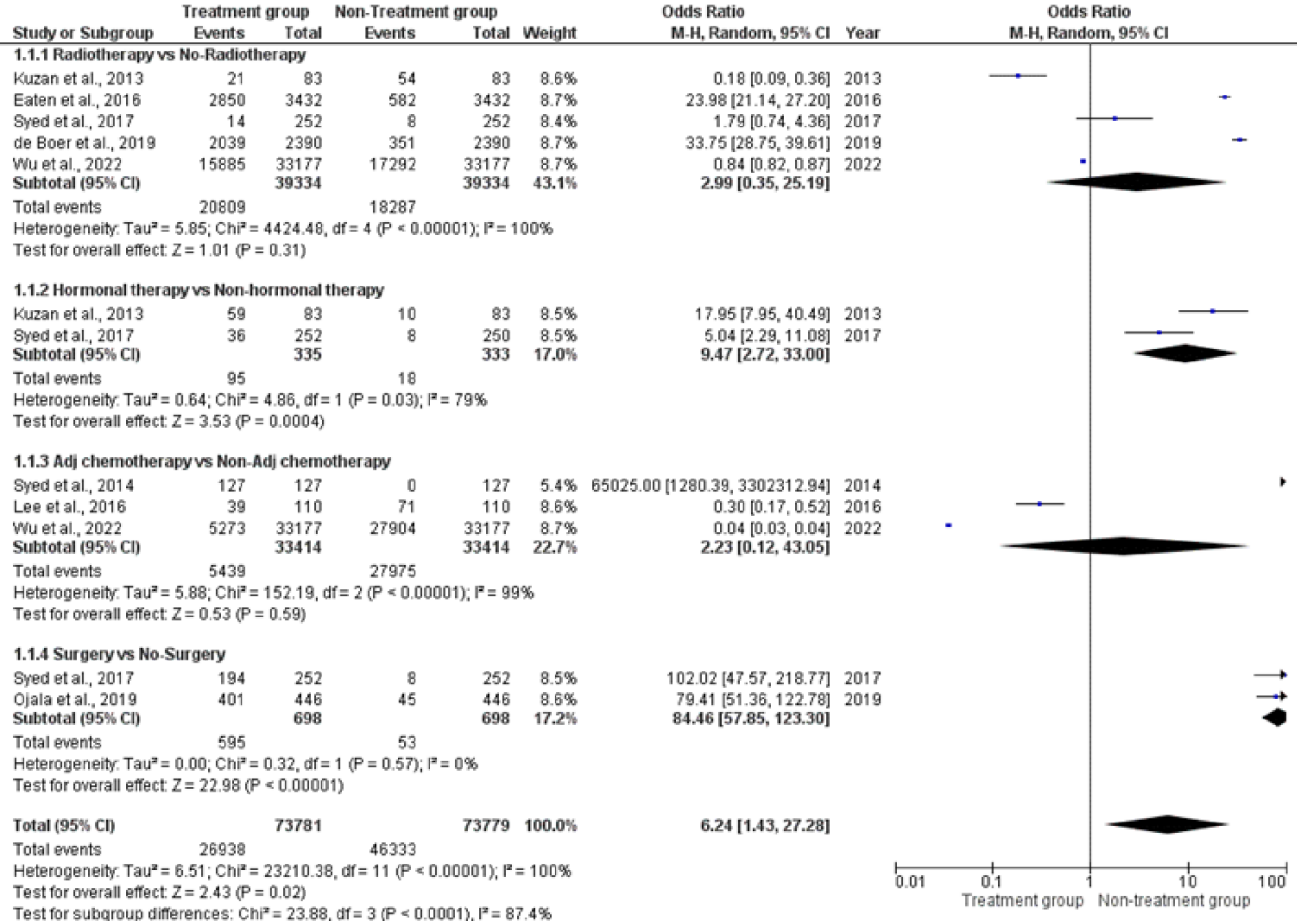
Forest plot for different treatment groups.

Sub-group analysis was performed (Fig. 2), and there was a difference between treatment and non-treatment groups. There was a significant difference with use of radiotherapy (OR=2.99, 95% CI 0.35-25.19, p<0.00001; *I^2^*=100%), with hormonal therapy (OR=9.47, 95% CI 0.72-33, p=0.03; *I^2^*=79%) and for Adj chemotherapy (OR=2.23, 95% CI 0.12-43.05, p=0.00001; *I^2^*=99%) while there was non-significant different in surgery subgroup (OR=84.46, 95% CI 57.85-123.23, p=0.57; *I^2^*=0%). Overall, there was 100% heterogeneity (p<0.00001) with OR=6.24, 95% CI 1.43-27.28 (Fig. 2).

### Quality assessment

The MMAT evaluation tool was used to evaluate the study's methodology. The quality of the selected research was consistently good (all values >3) (Supplementary Table 2).

## DISCUSSION

Among females, BC is the most prevalent form, and it is also the largest cause of cancer-related mortality globally. The availability of solid clinical and pathological prognostic, and predictive indications that can guide patient decision-making and therapy options are critical to the effective management of BC [67]. Over the past decade, an assessment of BC studies published in international journals shows that being older is strongly associated with an increased risk of acquiring BC. It is critical to remember that BC in older women has unique clinic-pathological features from the illness in younger women [68]. Despite the abundance of published material on breast cancer therapy, very few research articles have investigated the possibility of correlations between molecular surrogates and older women. Following the completion of a literature analysis that included the years from 2013 to 2023, we provided an updated summary of the staging, molecular surrogates, and therapy choices for BC in women > 70 years.

In the present study, important molecular surrogates for BC were identified such as ER, PR, Hormone receptor+, HER2 and Ki-67 are useful for diagnosis and prognosis of BC and useful for determining which therapy options is the best for each patient. A question arises, which is why most of the studies reported these most common molecular surrogates. The probable explanation, especially for the ER and PR, can be that it has been known for long that estrogen has a crucial stimulatory role in both the development and progression of BC [69]. Both processes are known to be influenced by estrogen. In addition, ER is considered one of the most crucial prognostic indicators in BC. Meanwhile, it is a member of a family of nuclear hormone receptors that perform the function of a transcription factor [70]. It is quite plausible that the PR has a role in the development of breast cancer, and it is possible that postmenopausal women are the ones who are most affected by its effects [71]. It is believed that the ER is responsible for regulating PR expression, and the existence of PR expression is seen as evidence of a well-functioning estrogen-ER axis [72]. In most of the occurrences, expression of PR corresponds with ER expression, therefore from a purely practical basis. A strong PR expression in the absence of ER may demand further testing. In situations of early BC, the existence of PR expression is significant from a prognostic perspective, with patients that are both PR and PR positive having the best result [73]. At the current time, standard practice involves monitoring ER and PR receptor expression. At the same time, HER2 (positive/negative) was also the most reported predictor in the present study, as HER2 gene overexpression is a strong predictive indication, which is also crucial to considered. However, HER-2 low is also used as a new prognostic predictor in the authors' clinical practice. Invasive breast carcinomas, including primary and metastatic, are now routinely tested for HER2 protein overexpression using an immune-histochemical assay. Amplification of the HER2/neu gene is the usual cause of the HER2 protein being overproduced as a secondary effect. Similarly, Ki67 was shown to be linked with prognosis, overall survival and disease-free survival in meta-analyses conducted by Azambuja, Cardoso [74] and Stuart-Harris, Caldas [75]. Meanwhile, most important, advance surrogates like Oncotype Dx and Mamaprint were not reported in the included studies, which may be due to the strict inclusion criteria. One other reason may be the limited research conducted on these advanced molecular surrogates. Substantially relevant is to align the scientific, clinical, and educational progress with the international health policy priorities [76].

**Table 5:** Conclusions and limitations of included studies.

Ref.	Deaths (%)	Conclusion	Limitations
[54]	24.09	Low fatality rates were found despite undertreating most patients.	NA
[57]	32	Endocrine therapy controlled operable BC in older adults and was well tolerated. Compliance was a major issue.	NA
[53]	NA	Older breast cancer patients behave differently and undergo various therapies.	Study design, small sample size.
[58]	29.40	Young patients had more aggressive illness, worsening DFS. Nonetheless, elderly TN patients have poorer DFS and DSS, suggesting that chemotherapy should not be avoided due to age.	Single center study, Study design, Missing data such comorbidities.
[59]	18.40	80-year-olds had poorer DFS and OS.	NA
[61]	44.30	Brazilian women over 80 with breast cancer survived surgery and hormone therapy.	Small sample size, Study design, missing data such as Ki-67 marker
[63]	NA	It was found that older patients (above 70 years) receive less systemic and local treatment than younger patients (50-69 years).	NA
[64]	48.60	Poor biological and clinical outcomes were seen in geriatric patients with early operable primary breast cancer that lacked the estrogen receptor. Surgery is the standard method for initial care.	NA
[52]	20	Breast cancer patients aged 70-79 received more aggressive therapies, suggesting that treatment choice in patients over 80 years old was likely dependent on age rather than tumor features.	Small sample size, study design, comorbid disease on treatment choice could not be clearly detected.
[62]	0.41	Even in hospitals with limited radiation utilization, locoregional recurrence risk was modest despite 39.3% endocrine treatment. This supports omitting radiation in T1-2N0 breast cancer patients over 75.	Low event rate, residual confounding,
[56]	NA	Adherent endocrine therapy did not significantly affect breast cancer-specific or overall mortality in older women with localized ER-positive breast cancer.	Study design and missing data
[60]	NA	Elderly Korean women have more aggressive clinicopathological and biochemical aspects of breast cancer than all Korean women or elderly women worldwide.	Selection bias, Missing data, small sample size, generalizability.
[55]	16.3 Chemo group, 18.39 non-chemo group	Chemotherapy may improve elderly breast cancer prognosis, especially in chemotherapy-responsive subpopulations.	Missing data

In the present study, four subtypes, such as luminal A and B, HER2-enriched and Triple negative, were identified. These discoveries agree with the results of Perou *et al.* [77], who used gene expression microarray data on a sample of breast tumors to identify four molecular subtypes of BC: Luminal, HER2-enriched, Normal Breast-like and Basal-like. Additional research made it possible to subdivide the Luminal group into two subgroups, which were designated as Luminal A and B [78]. Meanwhile, it was also shown that with the incidence of luminal (A, B, and A+B) cancers increased with age (p<0.01), but the proportion of basal-like tumors decreased (p<0.0001) [79]. Similarly, the molecular subtype luminal A is frequently linked to a better prognosis and generally less benefits from chemotherapy [80, 81]. Both characteristics point to a more favorable outcome for the patient. Compared to the other molecular subtypes, this subtype is known to have a more sedentary trajectory and a slower evolution over time [82]. In addition, having a positive status for hormone receptors is not only an indicator of an improved prognosis, but also a sign of how well a patient would respond to endocrine treatment [83]. This comprehensive geriatric approach improves survival outcomes in older adults [84].

Staging for BC is essential because it provides crucial information about the extent and severity of the disease, which helps doctors determine the best course of treatment for the patient. Staging is the process of classifying cancer based on its size, whether it has spread to nearby lymph nodes, and whether it has spread to other parts of the body. In the present study, there were five of 4 BC stages (0-IV) identified, and most of the patients had stage II and III BC as well as Grade II and III. In addition, BC spread to N0-N3, which may be due to delay reporting and under-report worrisome symptoms and lesions to their physicians. This may lead to a later diagnosis of BC at a more advanced stage, with presumably fewer treatment choices and worse results [42]. Our findings are in line with another study and revealed that diagnosis of stage II or III BC was more prevalent (39.2%) among individuals aged >80 [85]. Another study also supports our findings that elder women usually had stage II or III BC [86]. One may ask the reason why stage II and III BC is more prevalent in the present study. This may be due to some contributing factors such as screening, comorbidities, delayed screening or treatment or may aggressive tumor characteristics. However, it is crucial to remember that every instance of BC is distinct and may have a different set of underlying causes. Nevertheless, due to the patients or the physician preferences, we acknowledge that older patients are less frequently included in clinical trials [87]. So, it is critical that all women, even older women, get earlier adequate screening, diagnosis, and treatment for breast cancer.

Treatment choices for BC diagnosed at an early stage must weigh the benefits of reduced recurrence risk from certain medicines against the risks of treatment-related toxicity, which may be worsened in older patients due to physiological decline or comorbidities [88]. In the present study, patients undergoing a variety of treatments were included in all the papers that were considered for this assessment. These treatments included surgery, radiation, chemotherapy, hormonal and endocrine therapy, adjuvant therapy, and neoadjuvant therapy. In most of the research, surgical procedures were used to treat individuals diagnosed with BC (Table 4). We can inquire into the reason why most of the studies that reported surgical procedures are the best for elderly BC. The possible justification can be that both OS and BCSS were higher in older individuals who had surgical treatment [89]. The illness was well controlled both locally and regionally, most likely due to an increased incidence of surgical therapy. This elderly patient group seemed to be a good candidate for surgical therapy, which appeared safe [89]. Though, according to the sub-group analysis in the present study, there was a difference between treatment and non-treatment groups as there was the significant difference (p<0.01), with use of radiotherapy, with hormonal therapy. However, for Adj chemotherapy, there was non-significant different (p>0.01) in surgery subgroup (Figure 2). The reason for a non-significant difference between surgery and non-surgery in the present study might be the very limited number of research articles addressing surgery as the best option, and without the required data for meta-analysis. In contrast, the study conducted by Morgan [90] revealed that surgery for BC is entirely risk-free for patients > 70 years, and there is no mortality rate associated with the procedure among the 2816 patients who underwent the surgery. However, when dealing with older patients, the therapeutic approach must also consider their life expectancy, and other conditions that may endanger their well-being. The physical state categorization developed by the American Society of Anesthesiologists (ASA) may be used by the surgeon or the anesthesiologist to determine whether the surgical operation or the anesthetic will be safe for the patient [91]. When assessing the efficacy of breast cancer treatments, both OS and DFS are crucial metrics to consider as in the present study patients showed good OS and DFS rate (Table 4). However, these are not the only metrics that should be considered when deciding which treatment option is best for a certain patient. Other considerations, such as QoL, side effects from therapy, and personal preferences, play a relevant role.

The present investigation has several limitations. Firstly, due to the paucity of information on Ki67 expression, Dx, Mamaprint, and other comprehensive molecular markers. Maybe it justifies the fact that almost none of the included study defines Dx and Mamaprint as a predictor for BC, but we are only able to classify the patients into one of the four IHC-defined breast cancer subtypes. Secondly, we have only included articles in English to avoid language biasness. So, we may have missed data regarding other molecular surrogates (Dx, Mamaprint) or any other advanced biomarkers. More data on the impact of regular mammography screening on quality-of-life outcomes, morbidity outcomes, and mortality outcomes, as well as the usage of current imaging technology and BC treatments, would be beneficial. Data for specific groups of women, such as older women, or groups based on race or ethnicity, access to screening, or the prevalence of co-morbidities, may help to enhance screening methods. The examples include older women. Despite the lack of conclusive research on how new technologies like tomosynthesis affect screening results, their usage is expanding rapidly in developed countries. While several international scientific societies and journals draft guidance to the older adults with cancer inclusion, there are numerous regulatory global initiatives to give such a vulnerable group of patients access to efficient clinical trials [92].

**Table 6:** Characteristics of breast Cancer in the elderly.

Ref	Invasive Ductal Carcinoma (%)	Invasive Lobular Carcinoma (%)	Other types (%)	IHC				Subtype				Grade				Stage			
				ER	PR	HER2	Ki67	Luminal A	Luminal B	Triple negative	HER 2-positive	1	2	3	0	I	II	III	IV
**[54]**	70	8	20	73	70	NA	NA	NA	NA	NA	NA	16	38	16	NA	39		32	
**[57]**	NA	NA	NA	0	52.6	10.5	NA	NA	NA	NA	NA	NA	NA	NA	NA	10.5	36.8 IIa, 26.3 IIb	10.5 IIIa, 15.9 IIIb	0
**[53]**	75.3	8.5	11.5	75	67.3	NA	NA	NA	NA	NA	NA	NA	NA	NA	NA	NA	NA	NA	NA
**[58]**	72.8	11.8	15.4	NA	NA	12.6	NA	49	0.333	11.7	6	15.8	39.8	44.4	NA	32.2	40.1	19.9	7.8
**[59]**	68.9	11.4	5.8	75.1	66.9	18.9	NA	NA	NA	NA	NA	33.4	37.2	29.4	15.5	50.1	31.9	2.5	NA
**[61]**	74.3	Unclear	Unclear	76.9	65.2	13	NA	75.7		20.3	NA	NA	46.9	36.7	15.4		47.7	33.8	3.1
**[63]**	NA	NA	NA	87.6	77.4	16.1	NA	51	25.8	NA	16.1	14.6	63.5	22	NA	NA	NA	NA	NA
**[64]**	84.2	2.5	10.5	NA	26.8	16.9	NA	NA	NA	NA	NA	4.6	45.4	50	NA	NA	NA	NA	NA
**[52]**	65.5	9.2	25.2	79	70.2	14.3	NA	NA	NA	12.1	NA	16.4	41.6	26.9	5	24.8	42.9	27.3	NA
**[62]**	NA	NA	NA	NA	NA	NA	NA	NA	NA	NA	NA	Radiotherapy group=30.4; No radiotherapy group=32.2	Radiotherapy group=48.8; No radiotherapy group=42	Radiotherapy group=20.8; No radiotherapy group=25.8	NA	NA	NA	NA	NA
**[56]**	NA	NA	NA	NA	81.9	NA	NA	NA	NA	NA	NA	20.83	43.65	17.39	NA	NA	NA	NA	NA
**[60]**	79.1	2.3	63.2	63.2	48.3	21.8	NA	21.8	10.3	NA	NA	Unclear			11.5	29.9	35.6	14.9	8
**[55]**	NA	NA	87.5	87.5	76	10	NA	81.1	7.1	8.9	2.9	29	46.9	24.1	NA	57.7	33.2	9.2	NA
**Average**	73.76	7.67	12.63	80.06	65.49	14.9	NA	55.72	19.13	13.25	8.33	18.83	44.77	29.65	11.85	34.89	38.57	19.94	4.73

TNM (%)												Neoadjuvant Hormontherapy (%)		Neoadjuvant Chemotherapy (%)	
T					N					M					
Tis	T1	T2	T3	T4	N0	N1	N2	N3	N4	M0	M1	Yes	No	Yes	No
**NA**	16	41	13	12	30	21	11	5	NA	NA	NA	NA	NA	NA	NA
**NA**	NA	NA	NA	NA	NA	NA	NA	NA	NA	NA	NA	NA	NA	NA	NA
**NA**	NA	NA	NA	NA	NA	NA	NA	NA	NA	NA	NA	NA	NA	NA	NA
**NA**	NA	NA	NA	NA	NA	NA	NA	NA	NA	NA	NA	NA	NA	NA	NA
**15.5**	64.5	31.4	4.1	NA	NA	NA	NA	NA	NA	NA	NA	NA	NA	2.36	NA
**16.9**		47.7	13.8	12.5	NA	NA	NA	NA	NA	NA	NA	NA	NA	NA	NA
**NA**	NA	NA	NA	NA	NA	NA	NA	NA	NA	NA	NA	NA	NA	NA	NA
**NA**	NA	NA	NA	NA	NA	NA	NA	NA	NA	NA	NA	NA	NA	NA	NA
**NA**	35.3	46.2	8.4	8	47.9	23.9	7.1	7.1	NA	NA	NA	unclear 10.5			
**NA**	Radiotherapy group=71.1; Non radiotherapy group=60.7	Radiotherapy group=28.9; Non radiotherapy group=39.3	NA	NA	Unclear				NA	NA	NA	NA	NA	NA	NA
**NA**	NA	NA	NA	NA	NA	NA	NA	NA	NA	NA	NA	NA	NA	NA	NA
**11.5**	39.1	35.6	6.9	6.9	64.4	17.2	6.9	11.5	NA	92	8	NA	NA	NA	NA
**0**	63.7	29	4.6	2.6	77	17.1	3.8	2.2	NA	NA	NA	NA	NA	NA	NA
**10.98**	43.72	38.48	8.47	8.4	54.83	19.8	8.48	6.45	NA	92	NA	NA	NA	0.02	NA

Surgery		Adjuvant Hormonotherapy (%)		Adjuvant Chemotherapy (%)		Radiotherapy		Treatment refusal (%)	Treated in Specialized Geriatric Center	ECOG status	Geriatric 8 Assessment	Vulnerable Elderly Survey-13
Mastectomy	Breast conserving surgery	Yes	No	Yes	No	Yes	No					
										1 to 5		0-2
**10.00**	17	NA	NA	16.9	NA	25	75	NA	No	NA	NA	NA
**5.26**	NA	NA	NA	NA	NA	NA	NA	63.2	Unclear	NA	NA	NA
**71.00**	10.5	30.3	NA	69.8	NA	44.8	NA	NA	No	NA	NA	NA
**36.60**	NA	NA	NA	21.1	NA	79.1	NA	NA	No	NA	NA	NA
**NA**	Unclear	76.5	NA	NA	NA	77.43	NA	NA	Yes	NA	NA	NA
**NA**	25.71	NA	NA	NA	NA	34.28	65.71	NA	No	NA	NA	NA
**41.90**	52.9	NA	NA	NA	NA	26.2	73.8	NA	NA	NA	NA	NA
**77.00**	NA	NA	NA	NA	NA	5.6	NA	NA	No	NA	NA	NA
**NA**	32.8	Unclear 87.4				53.7	44.1	NA	No	NA	NA	NA
**NA**	NA	Unclear	Unclear	NA	NA	85.31	14.68	NA	No	NA	NA	NA
**NA**	NA	NA	NA	NA	NA	NA	NA	NA	No	NA	NA	NA
**24.10**	58.6	NA	NA	NA	NA	NA	NA	NA	No	NA	NA	NA
**12.30**	NA	NA	NA	NA	NA	47.9	52.1	NA	No	NA	NA	NA
**41.47**	32.92	15.53	NA	29	NA	40.3	54.2	63.2	7.7	NA	NA	NA

To resume it we enclose Table 6 with more specific data. The present study extensively investigates various facets of breast cancer treatment among elderly patients, encompassing molecular surrogates, subtypes, treatments, and outcomes. While the study provides valuable insights into the characteristics of the included research, it does not explicitly offer detailed findings or conclusions that directly establish surgery as particularly suitable for this age group. The study focuses on presenting a comprehensive overview of the collected data rather than delving into specific treatment rationales. Consequently, the assertion that surgery is advantageous for elderly breast cancer patients might be inferred from the broader context of the study's findings [1], but definitive support for this claim is not explicitly presented within the study itself. To address this gap, further research and literature could be explored to provide a more robust foundation for the conclusion regarding the suitability of surgery for elderly patients with breast cancer, compared to their younger counterparts [93].

While our investigation primarily focused on molecular surrogates, subtypes, and staging, it's worth noting that surgical interventions have emerged as a prominent consideration within the therapeutic landscape. The available data suggest that surgical procedures play a significant role in the management of breast cancer in older individuals. Various studies in our analysis indicated that surgical treatment, often in conjunction with other modalities, was associated with improved overall survival and controlled disease progression in this age group.

Through an examination of multiple research articles spanning the years 2013 to 2023, we revealed that surgery holds a significant place in disease control and survival enhancement among elderly breast cancer patients. Our findings indicate that surgical procedures were commonly employed and associated with favorable outcomes, including improved overall survival and disease-free survival rates. These results highlight the essential nature of surgical considerations in clinical decision-making, emphasizing its potential to extend life expectancy and enhance the quality of life for elderly breast cancer patients. As a result, our research holds the potential to influence clinical practices and treatment guidelines, advocating for a more pronounced incorporation of surgery within the therapeutic repertoire for elderly breast cancer patients. However, it's important to acknowledge that the suitability of surgery should be carefully evaluated in alignment with the unique molecular subtypes and clinical profiles of older patients, as our study findings have shown considerable variation among available therapies. Thus, while surgery is an integral component, its application must be balanced with considerations of patient-specific characteristics and preferences [1], in addition to the broader treatment options available.

No matter how old a patient is, in the current era, staging and molecular subtype are the most accurate predictor of the result. That’s why we updated an earlier description of BC staging, molecular surrogates, and treatment choices for women > 70 years based on a literature analysis encompassing the years of 2013 to 2023. We found that molecular markers such as ER, PR, Hormone receptor+, HER2, and Ki-67 are useful as prognostic indictors for BC. In addition, luminal A and B, HER2-enriched, and Triple negative were identified as the four major subtypes. There were four BC stages (0-IV) identified, and most of the patients had stage II and III BC as well as Grade II and III. In addition, BC spread to N0-N3. Possible treatments include surgery, radiation, chemotherapy, hormonal and endocrine therapy, adjuvant, and neoadjuvant therapy. Studies treating individuals with BC mostly used surgical procedures. Overall, there was substantial variation among available therapies. In general, patients showed good OS and DFS. However, the differences between the surgery groups were not statistically significant. Surgery should be carefully evaluated in older women with unfavorable molecular subtypes because it has the potential to considerably change disease-related prognosis. By learning more about the connection between tumor biology, age-specific therapy, and outcomes, we might perhaps refine our surgical approach. Breast cancer treatment is different worldwide because health systems and Global Geriatric Scales are different. Time to therapeutic decision, treatment and meetings should be critical to assist these patients, and that would bring clinical and economic benefits for patients and countries.

## Conclusion

The study's findings have far-reaching implications for transforming real-world clinical practices and treatment strategies in the realm of elderly breast cancer care. The identification of molecular surrogates such as ER, PR, Hormone receptor status, HER2, and Ki-67 as potent prognostic indicators offers a personalized avenue for tailoring treatments based on individual patient biology. This precision-focused approach holds the potential to amplify treatment responses while curtailing unnecessary interventions.

Furthermore, the delineation of distinct molecular subtypes like luminal A and B, HER2-enriched, and Triple-negative provides a foundation for targeted therapeutic approaches. By aligning treatment choices with the specific subtype, clinicians can potentially elevate treatment outcomes and mitigate adverse effects. The study's spotlight on the pivotal role of surgery in bolstering disease control and survival among elderly patients underscores the critical importance of surgical considerations in clinical decision-making.

The implications of our study extend into the realm of treatment guidelines, where the integration of molecular markers such as ER, PR, HER2, and Ki-67 can heighten the precision of diagnostic and prognostic evaluations. This, in turn, can drive personalized treatment recommendations that minimize unnecessary interventions while maximizing therapeutic benefits. The study's emphasis on the significance of luminal A and B subtypes, correlated with improved prognosis, advocates for a more robust incorporation of these factors into treatment decisions.

Additionally, identifying specific subgroups that might derive greater benefit from hormonal therapy than aggressive chemotherapy signifies a shift towards more targeted and less taxing interventions. In the meanwhile, some studies refer that overall, survival amongst older women was worse when receiving primary endocrine therapy [94].

Ultimately, this research holds the potential to catalyze a multidisciplinary approach in healthcare delivery. Collaboration among oncologists, surgeons, radiologists, and geriatric specialists becomes pivotal to ensure comprehensive care that addresses both the complexities of cancer and the unique needs of elderly patients [95]. These findings empower clinicians with evidence-backed insights while empowering patients to actively participate in shared decision-making throughout their treatment journey. As we chart the path ahead, the study's ramifications could translate into an enhanced quality of life, improved treatment outcomes, and a more streamlined healthcare experience for elderly breast cancer patients [96].

## Supplementary Materials

The Supplementary data can be found online at: www.aginganddisease.org/EN/10.14336/AD.2023.1002.



## References

[1] Cardoso F, Kyriakides S, Ohno S, Penault-Llorca F, Poortmans P, Rubio IT et al. (2019). ESMO Guidelines Committee. Early breast cancer: ESMO Clinical Practice Guidelines for diagnosis, treatment and follow-up. Ann Oncol, 30(10):1674. 31236598 10.1093/annonc/mdz189

[2] Nounou MI, ElAmrawy F, Ahmed N, Abdelraouf K, Goda S, Syed-Sha-Qhattal H (2015). Breast Cancer: Conventional Diagnosis and Treatment Modalities and Recent Patents and Technologies. Breast Cancer (Auckl),; 9(Suppl 2):17-34. 26462242 10.4137/BCBCR.S29420 PMC4589089

[3] Gülcan B, Oytun MG, Almuradova E, Doğu BB, Karaca B (2021). Breast Cancer in Women Aged 75 Years and Older. Eur J Geriatr Gerontol, 3(2):117-123.

[4] Kędzierawski P, Mężyk R (2021). Breast cancer in women aged 75 years and older - tumour characteristics and treatment options. Prz Menopauzalny, 20(1):14-20. 33935615 10.5114/pm.2021.104432 PMC8077806

[5] Łukasiewicz S, Czeczelewski M, Forma A, Baj J, Sitarz R, Stanisławek A. (2021). Breast Cancer—Epidemiology, Risk Factors, Classification, Prognostic Markers, and Current Treatment Strategies—An Updated Review. Cancers 2021, 13, 4287. 10.3390/cancers13174287. PMC8428369 34503097

[6] Giaquinto AN, Sung H, Miller KD, Kramer JL, Newman LA, Minihan A et al. (2022). Breast Cancer Statistics, 2022. CA Cancer J Clin, 72(6):524-541. 36190501 10.3322/caac.21754

[7] Joseph K, Zebak S, Alba V, Mah K, Au C, Vos L, Ghosh S, et al. (2021). Adjuvant breast radiotherapy, endocrine therapy, or both after breast conserving surgery in older women with low-risk breast cancer: Results from a population-based study. Radiother Oncol, 154:93-100. 32941956 10.1016/j.radonc.2020.09.017

[8] Mott NM, Markovitz NH, Wang T, Hughes TM, Pilewskie M, Jagsi R, et al. (2023). Avoiding Overtreatment of Women ≥70 With Early-Stage Breast Cancer: A Provider-Level Deimplementation Strategy. J Surg Res, 284:124-130. 36566589 10.1016/j.jss.2022.11.072

[9] Lee CS, Moy L, Joe BN, Sickles EA, Niell BL (2018). Screening for Breast Cancer in Women Age 75 Years and Older. AJR Am J Roentgenol, 210(2):256-263. 29112471 10.2214/AJR.17.18705

[10] Oeffinger KC, Fontham ET, Etzioni R, Herzig A, Michaelson JS, Shih YC, et al. (2015). American Cancer Society. Breast Cancer Screening for Women at Average Risk: 2015 Guideline Update From the American Cancer Society. JAMA, 314(15): 1599-614. 26501536 10.1001/jama.2015.12783 PMC4831582

[11] Walter LC, Schonberg MA (2014). Screening mammography in older women: a review. JAMA, 311(13):1336-47. 24691609 10.1001/jama.2014.2834 PMC4391705

[12] Qaseem A, Lin JS, Mustafa RA, Horwitch CA, Wilt TJ, Clinical Guidelines Committee of the American College of Physicians et al. (2019). Screening for Breast Cancer in Average-Risk Women: A Guidance Statement From the American College of Physicians. Ann Intern Med, 170(8):547-560. 30959525 10.7326/M18-2147

[13] Siu A(2016). U.S. Preventive Services Task Force. Screening for Breast Cancer: U.S. Preventive Services Task Force Recommendation Statement. Ann Intern Med. 2016 Feb 16;164(4):279-96. doi: 10.7326/M15-2886. Epub 2016 Jan 12. Erratum in: Ann Intern Med. 2016 Mar 15;164(6):448. PMID: 26757170. 26757170

[14] Lee CS, Monticciolo DL, Moy L (2020). Screening Guidelines Update for Average-Risk and High-Risk Women. AJR Am J Roentgenol, 214(2):316-323. 31714845 10.2214/AJR.19.22205

[15] Bevers T, Helvie M, Bonaccio E, Calhoun K, Daly M, Farrar W et al. (2018). Breast Cancer Screening and Diagnosis, Version 3. NCCN Clinical Practice Guidelines in Oncology. Journal of the National Comprehensive Cancer Network J Natl Compr Canc Netw, 16(11), 1362-1389. doi.org/10.6004/jnccn.2018.0083. 30442736

[16] Bevers T, Niell B, Baker J, Bennett D, Bonaccio E, Camp MS et al. (2023). NCCN Guidelines® Insights: Breast Cancer Screening and Diagnosis, Version 1. Journal of the National Comprehensive Cancer Network, 21(9), 900-909. doi.org/10.6004/jnccn.2023.0046. 37673117

[17] Cardoso F, Kyriakides S, Ohno S, Penault-Llorca F, Poortmans P, Rubio IT et al. (2019). Early breast cancer: ESMO Clinical Practice Guidelines for diagnosis, treatment and follow-up†. Ann Oncol, 30(8):1194-1220. 31161190 10.1093/annonc/mdz173

[18] Badgwell BD, Giordano SH, Duan ZZ, Fang S, Bedrosian I, Kuerer HM et al. (2008). Mammography before diagnosis among women age 80 years and older with breast cancer. J Clin Oncol, 26(15):2482-8. 18427152 10.1200/JCO.2007.12.8058

[19] Extermann M, Hernández-Favela CG, Soto Perez de Celis E, Kanesvaran R (2022). Global Aging and Cancer: Advancing Care Through Innovation. Am Soc Clin Oncol Educ Book, 42:1-8. 10.1200/EDBK_359154 35452248

[20] Ko G, Hallet J, Jerzak KJ, Chan W, Coburn N, Barabash V et al (2023). Low Rates of Medical Oncology Consultation for Older Women (≥ 70 Years) with Newly Diagnosed, Non-Metastatic Breast Cancer: A Population-Based Study. Ann Surg Oncol, 30(2):1054-1062. 36255513 10.1245/s10434-022-12640-8

[21] Schmidt H, Boese S, Lampe K, Jordan K, Fiedler E, Müller-Werdan U et al (2017). Trans sectoral care of geriatric cancer patients based on comprehensive geriatric assessment and patient-reported quality of life - Results of a multicenter study to develop and pilot test a patient-centered interdisciplinary care concept for geriatric oncology patients (PIVOG). J Geriatr Oncol, 8(4):262-270. 28533106 10.1016/j.jgo.2017.04.002

[22] Biganzoli L, Battisti NML, Wildiers H, McCartney A, Colloca G, Kunkler IH et al (2021). Updated recommendations regarding the management of older patients with breast cancer: a joint paper from the European Society of Breast Cancer Specialists (EUSOMA) and the International Society of Geriatric Oncology (SIOG). Lancet Oncol, 22(7):e327-e340. 34000244 10.1016/S1470-2045(20)30741-5

[23] Fallah P, Mulla N, Rose A, Panasci L (2021). 149P Can high Ki67 predict distant recurrence in early-stage breast cancer with low oncotype Dx score?. Ann Oncol, 32:S425-S426.

[24] Losk K, Freedman RA, Lin NU, Golshan M, Pochebit SM, Lester SC et al. (2017). Implementation of Surgeon-Initiated Gene Expression Profile Testing (Onco type DX) Among Patients With Early-Stage Breast Cancer to Reduce Delays in Chemotherapy Initiation. J Oncol Pract, 13(9):e815-e820. 28858535 10.1200/JOP.2017.023788

[25] Gradishar WJ, Moran MS, Abraham J, Aft R, Agnese D, Allison KH et al. (2022). Breast Cancer, Version 3.2022, NCCN Clinical Practice Guidelines in Oncology. J Natl Compr Canc Netw, 20(6):691-722. 35714673 10.6004/jnccn.2022.0030

[26] van de Vijver MJ, He YD, van't Veer LJ, Dai H, Hart AA, Voskuil DW, et al. (2002). A gene-expression signature as a predictor of survival in breast cancer. N Engl J Med, 347(25):1999-2009. 12490681 10.1056/NEJMoa021967

[27] Sotiriou C, Wirapati P, Loi S, Harris A, Fox S, Smeds J et al. (2006). Gene expression profiling in breast cancer: understanding the molecular basis of histologic grade to improve prognosis. J Natl Cancer Inst, 98(4):262-72. 16478745 10.1093/jnci/djj052

[28] Tang P, Tse GM (2016). Immunohistochemical Surrogates for Molecular Classification of Breast Carcinoma: A 2015 Update. Arch Pathol Lab Med, 140(8):806-14. 27472239 10.5858/arpa.2015-0133-RA

[29] Majid RA, Hassan HA, Muhealdeen DN, Mohammed HA, Hughson MD (2017). Breast cancer in Iraq is associated with a unimodally distributed predominance of luminal type B over luminal type A surrogates from young to old age. BMC Womens Health, 17(1):27. 28388952 10.1186/s12905-017-0376-0 PMC5383947

[30] Klepin H, Mohile S, Hurria A (2009). Geriatric assessment in older patients with breast cancer. J Natl Compr Canc Netw, 7(2):226-36. 19200420 10.6004/jnccn.2009.0016 PMC4397965

[31] Tesarova P (2016). Specific Aspects of Breast Cancer Therapy of Elderly Women. Biomed Res Int, 2016:1381695. 27807536 10.1155/2016/1381695 PMC5078631

[32] Angarita FA, Zhang Y, Elmi M, Look Hong NJ (2020). Older women's experience with breast cancer treatment: A systematic review of qualitative literature. Breast, 54:293-302. 33242756 10.1016/j.breast.2020.11.009 PMC7695983

[33] Sun SX, Hollenbeak CS, Leung AM (2015). Deviation from the Standard of Care for Early Breast Cancer in the Elderly: What are the Consequences? Ann Surg Oncol, 22(8):2492-9. 25515198 10.1245/s10434-014-4290-5

[34] Kocaöz S, Korukluoğlu B, Parlak Ö, Doğan HT, Erdoğan F (2019). Comparison of clinicopathological features and treatments between pre- and postmenopausal female breast cancer patients - a retrospective study. Prz Menopauzalny, 18(2):68-73. 31485202 10.5114/pm.2019.85786 PMC6719638

[35] Kędzierawski P, Mężyk R (2021). Breast cancer in women aged 75 years and older - tumour characteristics and treatment options. Prz Menopauzalny, 20(1):14-20. 33935615 10.5114/pm.2021.104432 PMC8077806

[36] Biganzoli L, Wildiers H, Oakman C, Marotti L, Loibl S, Kunkler I, Reed M et al. (2012). Management of elderly patients with breast cancer: updated recommendations of the International Society of Geriatric Oncology (SIOG) and European Society of Breast Cancer Specialists (EUSOMA). Lancet Oncol, 13(4):e148-60. 22469125 10.1016/S1470-2045(11)70383-7

[37] Singh JC, Lichtman SM (2015). Effect of age on drug metabolism in women with breast cancer. Expert Opin Drug Metab Toxicol, 11(5):757-66. 25940027 10.1517/17425255.2015.1037277 PMC5057182

[38] Freedman RA, Vaz-Luis I, Barry WT, Lii H, Lin NU, Winer EP et al. (2014) Patterns of chemotherapy, toxicity, and short-term outcomes for older women receiving adjuvant trastuzumab-based therapy. Breast Cancer Res Treat, 145(2):491-501. 24756187 10.1007/s10549-014-2968-9

[39] Vaz-Luis I, Keating NL, Lin NU, Lii H, Winer EP, Freedman RA (2014). Duration and toxicity of adjuvant trastuzumab in older patients with early-stage breast cancer: a population-based study. J Clin Oncol, 32(9):927-34. 24516021 10.1200/JCO.2013.51.1261 PMC3948095

[40] McGuire A, Brown JA, Malone C, McLaughlin R, Kerin MJ (2015). Effects of age on the detection and management of breast cancer. Cancers (Basel), 7(2):908-29. 26010605 10.3390/cancers7020815 PMC4491690

[41] Andrew LS, Shelby S, Peter PY (2018). Timing of adjuvant chemotherapy administration for early breast cancer. J Clin Oncol, 36:30_suppl, 62-62.

[42] Tesarova P (2012). Breast cancer in the elderly-Should it be treated differently? Rep Pract Oncol Radiother, 18(1):26-33. 24381744 10.1016/j.rpor.2012.05.005 PMC3863252

[43] Spring LM, Gupta A, Reynolds KL, Gadd MA, Ellisen LW, Isakoff SJ et al. (2016). Neoadjuvant Endocrine Therapy for Estrogen Receptor-Positive Breast Cancer: A Systematic Review and Meta-analysis. JAMA Oncol, 2(11):1477-1486. 27367583 10.1001/jamaoncol.2016.1897 PMC5738656

[44] Barchiesi G, Mazzotta M, Krasniqi E, Pizzuti L, Marinelli D, Capomolla E et al. (2020) Neoadjuvant Endocrine Therapy in Breast Cancer: Current Knowledge and Future Perspectives. Int J Mol Sci, 21(10):3528. 32429381 10.3390/ijms21103528 PMC7278946

[45] O'Rourke K (2022). Study shows that patients older than 80 years benefit from immunotherapy. Cancer, 128(6):1155-1156. 35218210 10.1002/cncr.34138

[46] Prieto-Callejero B, Rivera F, Fagundo-Rivera J, Romero A, Romero-Martín M, Gómez-Salgado J et al. (2020). Relationship between chemotherapy-induced adverse reactions and health-related quality of life in patients with breast cancer. Medicine (Baltimore), 99(33):e21695. 32872042 10.1097/MD.0000000000021695 PMC7437745

[47] Hurria A, Togawa K, Mohile SG, Owusu C, Klepin HD, Gross CP et al. (2011). Predicting chemotherapy toxicity in older adults with cancer: a prospective multicenter study. J Clin Oncol, 29(25):3457-65. 21810685 10.1200/JCO.2011.34.7625 PMC3624700

[48] Rossi A, Colantuoni G, Maione P, Ferrara C, Airoma G, Barzelloni ML et al. (2005). Chemotherapy of breast cancer in the elderly. Curr Med Chem, 12(3):297-310. 15723620 10.2174/0929867053363261

[49] Page MJ, Moher D, McKenzie JE (2022). Introduction to preferred reporting items for systematic reviews and meta-analyses 2020 and implications for research synthesis methodologists. Res Synth Methods, 13(2):156-163. 34693644 10.1002/jrsm.1535

[50] Schardt C, Adams MB, Owens T, Keitz S, Fontelo P (2007). Utilization of the PICO framework to improve searching PubMed for clinical questions. BMC Med Inform Decis Mak, 15;7:16. 10.1186/1472-6947-7-16 PMC1904193 17573961

[51] Charette M, McKenna LG, Deschênes MF, Ha L, Merisier S, Lavoie P (2020). New graduate nurses' clinical competence: A mixed methods systematic review. J Adv Nurs, 76(11):2810-2829. 32869369 10.1111/jan.14487

[52] Hong QN, Gonzalez-Reyes A, Pluye P (2018). Improving the usefulness of a tool for appraising the quality of qualitative, quantitative and mixed methods studies, the Mixed Methods Appraisal Tool (MMAT). J Eval Clin Pract, 24(3):459-467. 29464873 10.1111/jep.12884

[53] Cumpston M, Li T, Page MJ, Chandler J, Welch VA, Higgins JP et al. (2019). Updated guidance for trusted systematic reviews: a new edition of the Cochrane Handbook for Systematic Reviews of Interventions. Cochrane Database Syst Rev, 10(10):ED000142. 31643080 10.1002/14651858.ED000142 PMC10284251

[54] Aytekin A, Karatas F, Sahin S, Erdem GU, Altundag K (2017). Clinicopathological features of patients with breast cancer aged 70 years or over. J BUON, 22(1):200-207. 28365955

[55] Inal A, Akman T, Yaman S, Ozturk SC, Geredeli C, Bilici M et al. (2014). Pathologic and clinical characteristics of elderly patients with breast cancer: a retrospective analysis of a multicenter study (Anatolian Society of Medical Oncology). Int Surg, 99(1):2-7. 24444261 10.9738/INTSURG-D-13-00010 PMC3897335

[56] Kuzan TY, Koca E, Dizdar O, Arslan C, Eren T, Yalcin S et al. (2013). Breast cancer in octogenarian women: clinical characteristics and outcome. J BUON, 18(2):328-34. 23818342

[57] Wu Y, Qi Y, Yang J, Yang R, Lui W, Huang Y et al. (2022). Effect of adjuvant chemotherapy on the survival outcomes of elderly breast cancer: A retrospective cohort study based on SEER database. J Evid Based Med, 15(4):354-364. 36524240 10.1111/jebm.12506 PMC10108030

[58] Yuan C, Xie Z, Bian J, Huo J, Daily K (2020). Outcomes of primary endocrine therapy in elderly women with stage I-III breast cancer: a SEER database analysis. Breast Cancer Res Treat, 180(3):819-827. 32172303 10.1007/s10549-020-05591-9

[59] Chan SW, Chan PM, Seah MD, Chen JJ, Tan EY (2014). Limiting the use of primary endocrine therapy in elderly women with breast cancer. Ann Acad Med Singapore, 43(9):469-72. 25341632

[60] Acevedo F, Camus M, Sanchez C (2015). Breast cancer at extreme ages--a comparative analysis in Chile. Asian Pac J Cancer Prev, 16(4):1455-61. 25743815 10.7314/apjcp.2015.16.4.1455

[61] Angarita FA, Chesney T, Elser C, Mulligan AM, McCready DR, Escallon J (2015). Treatment patterns of elderly breast cancer patients at two Canadian cancer centres. Eur J Surg Oncol, 41(5):625-34. 25727372 10.1016/j.ejso.2015.01.028

[62] Kim SJ, Park YM (2020). Breast cancer in elderly Korean women: clinicopathological and biological features. Breast Dis, 39(2):71-83. 32250285 10.3233/BD-190422

[63] Bello MA, Menezes RF, Silva B, da Silva Rde C, Cavalcanti RS, Moraes TD et al. (2016). Impact of Treatment Type on Overall Survival in Elderly Brazilian Women with Breast Cancer. Asian Pac J Cancer Prev, 17(10):4769-4774. 27893210 10.22034/APJCP.2016.17.10.4769 PMC5454630

[64] de Boer AZ, Bastiaannet E, de Glas NA, Marang-van de Mheen PJ, Dekkers OM, Siesling S et al. (2019). Effectiveness of radiotherapy after breast-conserving surgery in older patients with T1-2N0 breast cancer. Breast Cancer Res Treat, 178(3):637-645. 31451977 10.1007/s10549-019-05412-8 PMC6817758

[65] Inwald EC, Ortmann O, Koller M, Zeman F, Hofstädter F, Evert M et al. (2017). Screening-relevant age threshold of 70 years and older is a stronger determinant for the choice of adjuvant treatment in breast cancer patients than tumor biology. Breast Cancer Res Treat, 163(1):119-130. 28205042 10.1007/s10549-017-4151-6 PMC5387012

[66] Syed BM, Morgan D, Setty T, Green AR, Paish EC, Ellis IO et al. (2017). Oestrogen receptor negative early operable primary breast cancer in older women-Biological characteristics and long-term clinical outcome. PLoS One, 12(12):e0188528. 29284000 10.1371/journal.pone.0188528 PMC5746234

[67] Łukasiewicz S, Czeczelewski M, Forma A, Baj J, Sitarz R, Stanisławek A (2021). Breast Cancer-Epidemiology, Risk Factors, Classification, Prognostic Markers, and Current Treatment Strategies-An Updated Review. Cancers (Basel), 13(17):4287. 34503097 10.3390/cancers13174287 PMC8428369

[68] Diab SG, Elledge RM, Clark GM (2000). Tumor characteristics and clinical outcome of elderly women with breast cancer. J Natl Cancer Inst, 92(7):550-6. 10749910 10.1093/jnci/92.7.550

[69] Feng Y, Spezia M, Huang S, Yuan C, Zeng Z, Zhang L et al. (2018). Breast cancer development and progression: Risk factors, cancer stem cells, signaling pathways, genomics, and molecular pathogenesis. Genes Dis, 5(2):77-106. 30258937 10.1016/j.gendis.2018.05.001 PMC6147049

[70] Early Breast Cancer Trialists' Collaborative Group (EBCTCG); Davies C, Godwin J, Gray R, Clarke M, Cutter D, Darby S et al. (2011). Relevance of breast cancer hormone receptors and other factors to the efficacy of adjuvant tamoxifen: patient-level meta-analysis of randomised trials. Lancet, 378(9793):771-84. 21802721 10.1016/S0140-6736(11)60993-8 PMC3163848

[71] Chlebowski RT, Hendrix SL, Langer RD, Stefanick ML, Gass M, Lane D et al. (2003). Influence of estrogen plus progestin on breast cancer and mammography in healthy postmenopausal women: the Women's Health Initiative Randomized Trial. JAMA, 289(24):3243-53. 12824205 10.1001/jama.289.24.3243

[72] Clarke RB (2003). Steroid receptors and proliferation in the human breast. Steroids, 68(10-13):789-94. 14667969 10.1016/s0039-128x(03)00122-3

[73] Pichon MF, Pallud C, Brunet M, Milgrom E (1980). Relationship of presence of progesterone receptors to prognosis in early breast cancer. Cancer Res, 40(9):3357-60. 7427948

[74] de Azambuja E, Cardoso F, de Castro G Jr, Colozza M, Mano MS, Durbecq V et al. (2007). Ki-67 as prognostic marker in early breast cancer: a meta-analysis of published studies involving 12,155 patients. Br J Cancer, 96(10):1504-13. 17453008 10.1038/sj.bjc.6603756 PMC2359936

[75] Stuart-Harris R, Caldas C, Pinder SE, Pharoah P (2008). Proliferation markers and survival in early breast cancer: a systematic review and meta-analysis of 85 studies in 32,825 patients. Breast, 17(4):323-34. 18455396 10.1016/j.breast.2008.02.002

[76] Extermann M, Brain E, Canin B, Cherian MN, Cheung KL, de Glas N et al. (2021). Priorities for the global advancement of care for older adults with cancer: an update of the International Society of Geriatric Oncology Priorities Initiative. Lancet Oncol, 22(1):e29-e36. 33387502 10.1016/S1470-2045(20)30473-3

[77] Perou CM, Sørlie T, Eisen MB, van de Rijn M, Jeffrey SS, Rees CA et al. (2000). Molecular portraits of human breast tumours. Nature, 406(6797):747-52. 10963602 10.1038/35021093

[78] Prat A, Perou CM (2011). Deconstructing the molecular portraits of breast cancer. Mol Oncol, 5(1):5-23. 21147047 10.1016/j.molonc.2010.11.003 PMC5528267

[79] Jenkins EO, Deal AM, Anders CK, Prat A, Perou CM, Carey LA et al. (2014). Age-specific changes in intrinsic breast cancer subtypes: a focus on older women. Oncologist, 19(10):1076-83. 25142841 10.1634/theoncologist.2014-0184 PMC4200998

[80] Tsoutsou PG, Vozenin MC, Durham AD, Bourhis J (2016). How could breast cancer molecular features contribute to locoregional treatment decision making?. Crit Rev Oncol Hematol, 110:43-48. 28109404 10.1016/j.critrevonc.2016.12.006

[81] Sanpaolo P, Barbieri V, Genovesi D (2011). Prognostic value of breast cancer subtypes on breast cancer specific survival, distant metastases and local relapse rates in conservatively managed early stage breast cancer: a retrospective clinical study. Eur J Surg Oncol, 37(10):876-82. 21824742 10.1016/j.ejso.2011.07.001

[82] Jatoi I, Anderson WF, Jeong JH, Redmond CK. Breast cancer adjuvant therapy: time to consider its time-dependent effects. J Clin Oncol, 29(17):2301-4. 10.1200/JCO.2010.32.3550 PMC3107746 21555693

[83] van der Leij F, Elkhuizen PH, Bartelink H, van de Vijver MJ (2012). Predictive factors for local recurrence in breast cancer. Semin Radiat Oncol. 22(2):100-7. 22385917 10.1016/j.semradonc.2011.12.001

[84] Nishijima TF, Shimokawa M, Komoda M, Hanamura F, Okumura Y, Morita M et al. (2023). Survival in Older Japanese Adults With Advanced Cancer Before and After Implementation of a Geriatric Oncology Service. JCO Oncol Pract, 18:OP2200842. 10.1200/OP.22.00842 37200607

[85] Bertolo A, Rosso C, Voutsadakis IA (2020). Breast Cancer in Patients 80 Years-Old and Older. Eur J Breast Health, 16(3):208-212. 32656522 10.5152/ejbh.2020.5659 PMC7337909

[86] Reddy A, Mullapudi NA, Kabeer KK, Nimmagadda R, Radhakrishna S (2019). Treatment of elderly breast cancer patients in a breast center in India. Indian J Cancer, 56(1):45-49. 30950444 10.4103/ijc.IJC_237_18

[87] de Glas NA (2020). Geriatric Oncology: From Research to Clinical Practice. Cancers (Basel), 12(11):3279. 33167596 10.3390/cancers12113279 PMC7694457

[88] O'Connor T, Shinde A, Doan C, Katheria V, Hurria A (2013). Managing breast cancer in the older patient. Clin Adv Hematol Oncol, 11(6):341-7. 24472802 PMC3906632

[89] Ojala K, Meretoja TJ, Mattson J, Leidenius MHK (2019). Surgical treatment and prognosis of breast cancer in elderly - A population-based study. Eur J Surg Oncol, 45(6):956-962. 30691722 10.1016/j.ejso.2019.01.019

[90] Morgan JL, George J, Holmes G, Martin C, Reed MWR, Ward S et al. (2020). Bridging the Age Gap Trial Management Team. Breast cancer surgery in older women: outcomes of the Bridging Age Gap in Breast Cancer study. Br J Surg, 107(11):1468-1479. 32488911 10.1002/bjs.11617

[91] Keats AS (1978). The ASA classification of physical status--a recapitulation. Anesthesiol, 49(4):233-6. 10.1097/00000542-197810000-00001 697075

[92] Kanesvaran R, Mohile S, Soto-Perez-de-Celis E, Singh H (2020). The Globalization of Geriatric Oncology: From Data to Practice. Am Soc Clin Oncol Educ Book, 40:1-9. 10.1200/EDBK_279513 32347757

[93] Syed BM, Green AR, Nolan CC, Morgan DA, Ellis IO, Cheung KL (2014). Biological characteristics and clinical outcome of triple negative primary breast cancer in older women - comparison with their younger counterparts. PLoS One, 9(7):e100573. 24999743 10.1371/journal.pone.0100573 PMC4085072

[94] Chan KS, Chong MTH, Chia CLK, Cheung KL (2023). Revisiting primary endocrine therapy versus surgery in older women with breast cancer: meta-analysis. Br J Surg, 110(4):420-431. 36718056 10.1093/bjs/znac435

[95] Eaton BR, Jiang R, Torres MA, Kahn ST, Godette K, Lash TL et al. (2016). Benefit of adjuvant radiotherapy after breast-conserving therapy among elderly women with T1-T2N0 estrogen receptor-negative breast cancer. Cancer, 122(19):3059-68. 27328114 10.1002/cncr.30142 PMC5030146

[96] Lee HCh, Chen WY, Huang WN, Cheng KO, Tian YF, Ho ChH et al. (2016). Impact of Adjuvant Chemotherapy in Elderly Breast Patients in Taiwan, A Hospital-Based Study. Asian Pac J Cancer Prev, 17(10):4591-4597. 27892670 10.22034/APJCP.2016.17.10.4591 PMC5454603

