# Supplementary material for: Geriatric Breast Cancer: Staging, Molecular Surrogates, and Treatment. A Review & Meta-analysis
Source: Aging Dis. 2024 Aug 1;15(4):1602–18. doi: 10.14336/AD.2023.1002 (PMC11272193; doi:10.14336/AD.2023.1002)
Supplement: Supplementary file 1 [file AD-15-4-1602-S.pdf]

SUPPLEMENTARY DATA

Geriatric Breast Cancer: Staging, Molecular Surrogates, and Treatment. A Review & Meta-analysis

Vasco C. Fonseca, Zacharoula Sidiropoulou

© 2023. Fonseca VC. et al. Published online at http://www.aginganddisease.org/EN/10.14336/AD.2023.1002

SUPPLEMENTARY DATA

Supplementary Table 1. Search strategy for different databases.

Databases	Key terms
PubMed	(((Breast cancer[MeSH Terms]) AND (Older[Title/Abstract] OR Elder[Title/Abstract] OR Eldest[Title/Abstract] OR Elderly[Title/Abstract] OR Over 70 years[Title/Abstract])) AND (Molecular surrogates[Title/Abstract] OR HER2[Title/Abstract] OR ER[Title/Abstract] OR PR[Title/Abstract] OR Ki-67[Title/Abstract] OR EGFR[Title/Abstract] OR P53[Title/Abstract] OR Androgen receptor[Title/Abstract] OR Oncotype Dx[Title/Abstract] OR Mamaprint[Title/Abstract])) AND (Surgery[Title/Abstract] OR Chemotherapy[Title/Abstract] OR Hormonal therapy[Title/Abstract] OR Radiotherapy[Title/Abstract] OR Immunotherapy[Title/Abstract] OR Chemo-radiotherapy[Title/Abstract])
Scopus	("Breast cancer") AND ("Older" OR "Elder" OR "Eldest" OR "Elderly" OR "Over 70 years") AND ("Molecular surrogates" OR "HER2" OR "ER" OR "PR" OR "Ki-67" OR "EGFR" OR "P53" OR "Androgen receptor" OR "Oncotype Dx" OR "Mamaprint") OR ("Surgery" OR "Chemotherapy" OR "Hormonal therapy" OR "Radiotherapy" OR "Immunotherapy" OR "Chemo-radiotherapy")
Web of Sciences	((((TI=("Breast cancer")) AND TI=("Older" OR "Elder" OR "Eldest" OR "Elderly" OR "Over 70 years")) AND TI=("Molecular surrogates" OR "HER2" OR "ER" OR "PR" OR "Ki-67" OR "EGFR" OR "P53" OR "Androgen receptor" OR "Oncotype Dx" OR "Mamaprint"))) AND TI=("Surgery" OR "Chemotherapy" OR "Hormonal therapy" OR "Radiotherapy" OR "Immunotherapy" OR "Chemo-radiotherapy")
MEDLINE	("Breast cancer") AND ("Older" OR "Elder" OR "Eldest" OR "Elderly" OR "Over 70 years") AND ("Molecular surrogates" OR "HER2" OR "ER" OR "PR" OR "Ki-67" OR "EGFR" OR "P53" OR "Androgen receptor" OR "Oncotype Dx" OR "Mamaprint") OR ("Surgery" OR "Chemotherapy" OR "Hormonal therapy" OR "Radiotherapy" OR "Immunotherapy" OR "Chemo-radiotherapy")

Supplementary Table 2. Summary of Mixed Methods Appraisal Tool (MMAT) methodological quality assessment.

Ref.	Study design	MMAT Criteria for different studies				
		3.1	3.2	3.3	3.4	3.5
[54]	Retrospective	Y	Y	Y	Y	Y
[57]	Retrospective	Y	Y	Y	Y	Y
[53]	Retrospective	Y	Y	Y	Y	Y
[87]	Retrospective	Y	Y	Y	Y	Y
[58]	Retrospective	Y	Y	Y	C	Y
[59]	Retrospective chart review	Y	Y	Y	Y	Y
[61]	Retrospective	Y	Y	Y	Y	Y
[88]	Retrospective	Y	Y	Y	Y	Y
[89]	Retrospective	N	Y	Y	Y	Y
[63]	Retrospective	Y	Y	Y	Y	Y
[64]	Retrospective	Y	Y	Y	Y	Y
[52]	Retrospective	Y	Y	Y	Y	Y
[62]	Retrospective	Y	Y	Y	Y	Y
[84]	Retrospective	Y	Y	Y	N	Y
[56]	Retrospective	Y	Y	Y	Y	Y
[60]	Retrospective	Y	Y	Y	N	Y
[55]	Retrospective Cohort study	Y	Y	Y	Y	Y

© 2023. Fonseca VC. et al. Published online at http://www.aginganddisease.org/EN/10.14336/AD.2023.1002
